# Evidence that the population of quiescent bone marrow-residing very small embryonic/epiblast-like stem cells (VSELs) expands in response to neurotoxic treatment

**DOI:** 10.1111/jcmm.12315

**Published:** 2014-06-04

**Authors:** Katarzyna Grymula, Maciej Tarnowski, Katarzyna Piotrowska, Malwina Suszynska, Katarzyna Mierzejewska, Sylwia Borkowska, Katarzyna Fiedorowicz, Magda Kucia, Mariusz Z Ratajczak

**Affiliations:** a Department of Physiology, Pomeranian Medical UniversitySzczecin, Poland; b Stem Cell Institute at James Graham Brown Cancer Center, University of LouisvilleLouisville, KY, USA

**Keywords:** kainic acid, neurotoxicity, VSELs, stem cell mobilization

## Abstract

The concept that bone marrow (BM)-derived cells may participate in neural regeneration remains controversial, and the identity of the specific cell type(s) involved remains unknown. We recently reported that the adult murine BM contains a highly mobile population of Sca-1^+^Lin^−^CD45^−^ cells known as very small embryonic/epiblast-like stem cells (VSELs) that express several markers of pluripotency such as Oct-4. In the BM microenvironment, these cells are kept quiescent because of epigenetic modification of certain paternally imprinted genes. However, as reported, these cells can be mobilized in mice in an experimental model of stroke and express several genes involved in neurogenesis while circulating in peripheral blood (PB). In the current work, we employed a model of toxic brain damage, which is induced by administration of kainic acid, to see not only whether VSELs can be mobilized into PB in response to this neurotoxin, but, more importantly, whether they proliferate and expand in BM tissue. We report here for the first time that brain damage leads to activation and expansion of the BM pool of quiescent VSELs, which precedes their subsequent egress into PB. Harnessing these cells in neural tissue regeneration is currently one of the challenges in regenerative medicine.

## Introduction

The goal of regenerative medicine is to ameliorate irreversible destruction of brain tissue by harnessing the power of stem cells in the process of neurogenesis [1–3]. There are several clinical situations where stem cells could be employed to recover the normal function of the central nervous system (CNS). The most important examples are (*i*) stroke, (*ii*) traumatic brain injury, (*iii*) spinal cord injury and (*iv*) neurodegenerative disorders (Alzheimer's disease, Parkinsonism, amyotrophic lateral sclerosis and Huntington's disease). Thus far, the presence of stem cells has been clearly demonstrated at a few locations in the brain: in the subventricular zone (SVZ) of the lateral ventricles and olfactory bulb as well as in the subgranular zone (SGZ) of the dentate gyrus (DG) in the hippocampus [4–6].

Several types of stem cells, including mesenchymal stem cells (MSCs), haematopoietic stem cells (HSCs), as well as neural cells differentiated from embryonic stem cell lines, have been proposed as potential therapeutic vehicles [1,7]. Following this approach, our group found that murine bone marrow (BM) contains a mobile population of Sca-1^+^lin^−^CD45^−^ cells that express Oct-4^+^ known as very small embryonic-like stem cells (VSELs), which are mobilized into peripheral blood (PB) in a murine stroke model [8] as well as in patients suffering from stroke [9]. The number of VSELs in circulation also increases in other pathological situations related to organ/tissue damage such as heart infarct, sodium iodide-induced retinal damage, skin burns, as well as after pharmacological mobilization, for example, by administration of granulocyte colony-stimulating factor (G-CSF) [10–13]. Based on published data both in human and mouse, we propose that VSELs are population of epiblast/germline-derived stem cells and play an important role as an organ-residing reserve population of pluripotent/multipotent stem cells that give rise to stem cells already committed to particular organs and tissues [14–16]. These cells are kept quiescent by changes in expression of imprinted genes mainly involved in regulation of somatotropic signalling and cell cycle regulation [17,18]. Moreover, in appropriate *in vitro* models, murine and human VSELs have been demonstrated to differentiate into neurons and glial cells (astrocytes and oligodendrocytes) [19]. Based on observations that the number of circulating VSELs in PB increases in mice and humans after stroke [8,9], we envision that VSELs residing in adult tissues or mobilized into PB could be harnessed in regenerative medicine as a source of stem cells for neurogenesis and repair of the CNS.

In this study, we employed a model of toxic brain damage induced by the neurotoxin kainic acid (KA) [20] to see not only whether VSELs could be mobilized into PB in response to KA, but, more importantly, whether they proliferate and expand in response to neurotoxic damage in BM tissue. We report for the first time that brain damage leads to activation and expansion of the BM pool of VSELs as well as their specification into early neural progenitors. We envision that this step precedes their egress from BM into PB.

## Material and Methods

### Experimental animals and KA treatment

All experiments were performed on ninety 6–8-week-old male C57BL/6 mice that were divided into three experimental groups (based on the dose of KA) and one control group (Fig. S1A). Group A was treated with 8.5 mg/kg b.w., group B was treated with 15 mg/kg b.w. and group C was treated with 25 mg/kg b.w. in a single, subcutaneous injection of KA dissolved in saline. Each of the three groups was also divided into five subgroups, based on the time which past from injection to the dissection. Subgroup I was killed 6 hrs after injection, subgroup II was killed after 12 hrs, subgroup III after 24 hrs, subgroup IV after 48 hrs and subgroup V after 7 days. Five mice from control group were injected with saline only.

Based on the results obtained in the second phase of our experiment, we used ten C57BL/6 mice/group, which were injected with 25 mg/kg b.w. KA. Injections were performed five times during the 17 days of the experiment. KA-injected mice were given a bromodeoxiuridine (BrdU) dose of 50 mg/kg b.w. daily, while the control group was injected with BrdU and saline (Fig. S1B). After 17 days, mice were killed, and BM and PB samples and brain were harvested for analysis. Animal procedures were approved by the Local Ethics Committee and performed in accordance with guidelines for laboratory animal care. All efforts were made to minimize animal suffering and the number of animals used.

### Tissue preparation

At set time-points, brain, BM and PB samples were harvested. Briefly, brains were removed from the skulls and fixed in 10% buffered formalin for 24 hrs. After fixation, samples were dehydrated and embedded in paraffin blocks. Deparaffinized sections of the brain tissue (3 μm thick) were hydrated and stained with Mayer's haematoxylin and eosin (Sigma-Aldrich, St. Louis, MO, USA), according to the manufacturer protocol. After staining, sections were dehydrated in 95% and 99.8% alcohol, cleared with xylene and mounted with Roti® - Histokitt II mounting medium. Sections were observed under an Olympus IX81 inverted microscope (Olympus, Tokyo, Japan), and micrographs were collected with CellSense software (Olympus).

Samples for BM cell analysis were obtained by flushing femurs and tibiae with cold PBS containing 2% foetal bovine serum (FBS), L-glutamine and antibiotics. After isolation, the BM was depleted of erythrocytes by 15-minute incubations with ammonium chloride-containing lysing solution (BD Pharm Lyse, Lysing Buffer, Becton Dickinson, San Jose, CA, USA), passed through a 40-μm nylon mesh (Cell Strainer, BD Falcon, Becton Dickinson), centrifuged (300 × g, 4°C, 5 min.), and finally stained with antibodies, as described below. Mouse PB was collected in heparinized tubes from the vena cava during the dissection. Isolation of mononuclear cells (MNCs) was performed in an analogous way as BM-isolated cells.

### FACS-based analysis and sorting of VSELs and HSCs from murine BM and PB

Total nucleated cells (TNCs), which were obtained from BM and PB, were subsequently stained for CD45, haematopoietic lineage markers (Lineage [Lin]) and Sca-1 antigen for 30 min. in medium containing 2% foetal bovine serum. The following antimouse antibodies (BD Pharmingen, San Diego, CA, USA) were used for staining: rat anti-CD45 (allophycocyanin-Cy7, clone 30F11), anti-CD45R/B220 (PE, clone RA-6B2), anti-Gr-1 (PE, clone RB6-8 C5), anti-T-cell receptor-αβ (PE, clone H57-5970, anti-T-cell receptor-ɤδ (PE, clone GL3), anti-CD11b (PE, clone M1/70), anti-Ter119 (PE, clone TER-119) and anti-Ly-6A/E (also known as Sca-1, biotin, clone E13-161.7, with streptovidin conjugated to PE-Cy5). Cells were then washed, resuspended in RPMI-medium with 2% foetal bovine serum, and sorted with an Influx cell sorter (Becton Dickinson). Two populations were analysed: Lin^−^, Sca-1+, CD45^−^ (VSELs) and Lin^−^, Sca-1^+^, CD45^+^ (HSCs) [21].

### Real-time quantitative reverse transcription PCR (RQ-PCR)

Total RNA was isolated from cells harvested from BM and PB from experimental and control mice by employing the RNeasy Kit (Qiagen, Valencia, CA, USA). The RNA was reverse-transcribed with MultiScribe reverse transcriptase and oligo-dT primers (Applied Biosystems, Foster City, CA, USA). Quantitative assessment of mRNA levels was performed by real-time reverse transcriptase polymerase chain reaction (RT-PCR) on an ABI 7500 Fast instrument employing Power SyBR Green PCR Master Mix reagent. Real-time conditions were as follows: 95°C (15 sec.) followed by 40 cycles at 95°C (15 sec.) and 60°C (1 min.). According to melting point analysis, only one PCR product was amplified under these conditions. The relative quantity of target, normalized to the endogenous control β-2 microglobulin gene and relative to a calibrator, is expressed as fold change (2^−ΔΔCt^), where ΔCt = (Ct of target gene) – (Ct of the endogenous control gene, β-2 microglobulin), and ΔΔCt = (ΔCt of target gene) – (ΔCt of calibrator for the target gene).

The following primer pairs were used: β2-microglobin: 5′-CAT ACG CCT GCA GAG TTA AGC A-3′ (forward), 5′-GAT CAC ATG TCT CGA TCC CAG TAG-3′ (reverse); Oct-4: 5′-TTC TCA ATG CTA GTT CGC TTT CTC T-3′ (forward), 5′-ACC TTC AGG AGA TAT GCA AAT CG-3′ (reverse); Nanog: 5′-TTT TCA GAA ATC CCT TCC CTC G-3′ (forward), 5′-CGT TCC CAG AAT TCG ATG CTT-3′ (reverse); Sox2: 5′-GCG GAG TGG AAA CTT TTG TCC-3′ (forward), 5′-GGG AAG CGT GTA CTT ATC CTT CT-3′ (reverse); Rex1: 5′-AGA TGG CTT CCC TGA CGG ATA-3′ (forward), 5′-CCT CCA AGC TTT CGA AGG ATT T-3′ (reverse); Nes: 5′-TTG AGG CCT CCA GAA GAA GA-3′ (forward), 5′-GCC ATC TGC TCC TCT TTC AC-3′ (reverse); Olig1: 5′-ACG TCG TAG CGC AGG CTT AT-3′ (forward), 5′-CGC CCA ACT CCG CTT ACT T-3′ (reverse); Olig2: 5′-GGG AGG CGC CAT TGT ACA-3′ (forward), 5′-GTG CAG GCA GGA AGT TCC A-3′ (reverse); GFAP: 5′-GGA GCT CAA TGA CCG CTT TG-3′ (forward), 5′-TCC AGG AAG CGG ACC TTC TC-3′ (reverse), β3-tubulin: 5′-CCC AGC GGC AAC TAT GTA GG-3′ (forward), 5′-CCA GAC CGA ACA CTG TCC A-3′ (reverse).

In RT-PCR experiments to study Oct4A expression, we used primers by Mizuno and Kosaka [22] 5′-CCCCAATGCCGTGAAGTTGGAGAAGGT-3′ (forward), 5′-TCTCTAGCCCAAGCT GATTGGCGATGTG-3′ (reverse), while for Nanog 5′-CTGGGAACGCCTCATCAA-3′ (forward), 5′-CATCTTCTGCTTCCTGGCAA-3′ (reverse) and Nestin, we used primers from the above list, with GAPDH serving as positive control.

### Immunohistochemical staining of FACS-sorted VSELs from BM and PB

Lin^−^/Sca-1^+^/CD45^−^ cells (VSELs) were sorted, plated on 22-mm diameter plates coated with poly-L-lysine (P9155, Sigma-Aldrich, St. Louis, MO, USA), and incubated for 24 hrs. Subsequently, cells were fixed in 3.5% paraformaldehyde for 15 min., permeabilized by 0.1% Triton X100, washed in PBS, and pre-blocked with 2.5% BSA for 2.5 hrs at RT to avoid nonspecific binding of antibodies. The cells were then stained overnight at 4°C for Oct-4 (mAb, 1:150, MAB4419; Millipore, Billerica, MA, USA) and 1 hr at 37°C with an antibody to nestin (mAb, 1:150, MAB6767; Abnova, Atlanta, GA, USA). Appropriate secondary Alexa Fluor 488 goat antimouse IgG and Alexa Fluor 594 goat anti-rat IgG antibodies were used (1:500; Molecular Probes, Grand Island, NY, USA, A11001 and A11007 respectively) at 37°C for 1 hr. Nuclei were stained with DAPI (Invitrogen, Foster City, CA, USA) for 20 min. at 37°C. All images were captured with an *Olympus FV1000* confocal *microscope*.

### *In vitro* clonogenic assays

The growth of murine HSPCs isolated from BM and PB was evaluated by *in vitro* clonogenic assay, as described previously [23]. Briefly, 2 × 10^5^ BM- derived and 4 × 10^5^ PB-derived cells were resuspended in 0.4 ml of RPMI-1640 medium and mixed with 1.8 ml of MethoCult HCC-4230 methylcellulose medium (StemCell Technologies Inc., Vancouver, Canada), supplemented with L-glutamine and antibiotics. Specific murine recombinant growth factors (all from R&D Systems, Minneapolis, MN, USA) were added. To stimulate granulocyte-macrophage colony-forming units, IL-3 (20 U/ml), SCF (10 ng/ml) and GM-CSF (5 ng/ml) were used. EPO (5 U/ml), SCF (10 ng/ml) and IL-3 (20 U/ml) were used to stimulate erythrocyte burst-forming units (BFU-E). The colonies were counted under an inverted microscope after 7–10 days of culture. Each clonogenic assay was performed in quadruplicate.

### Statistic analysis

All data were analysed using Microsoft Excel 2007 and Statistica version 7.1 software. Mostly results are presented as mean ± standard error of the mean. In case of BrdU experiment, results are also presented as median values. The Mann–Whitney U and Student's *t*-tests were used, and statistical significance was defined as *P* < 0.05.

## Results

### Exposure to KA in mice results in damage to the brain

To establish an appropriate dose of KA, mice were injected with increasing doses of this neurotoxin (8.5–25 mg/kg b.w.), and histological analysis of brain sections was performed at 6, 12, 24, 48 and 168 hrs (7 days) after injection. We observed that, as expected, even a single injection of KA (15 or 25 mg/kg b.w.) may lead to damage to the cortex, behavioural changes and epileptic attacks with limb contractures in experimental animals (data not shown). We observed extensive damage to the brain when KA was applied at the highest dose (25 mg/kg b.w.) and injected five times in 3-day intervals. Figure S2 shows representative morphological changes in the hippocampus of treated animals, as reflected by a visible loss of cells in the DG.

### Exposure to neural toxic effects of KA results in expansion and subsequent mobilization into PB of BM-residing VSELs

**Fig. 1:**
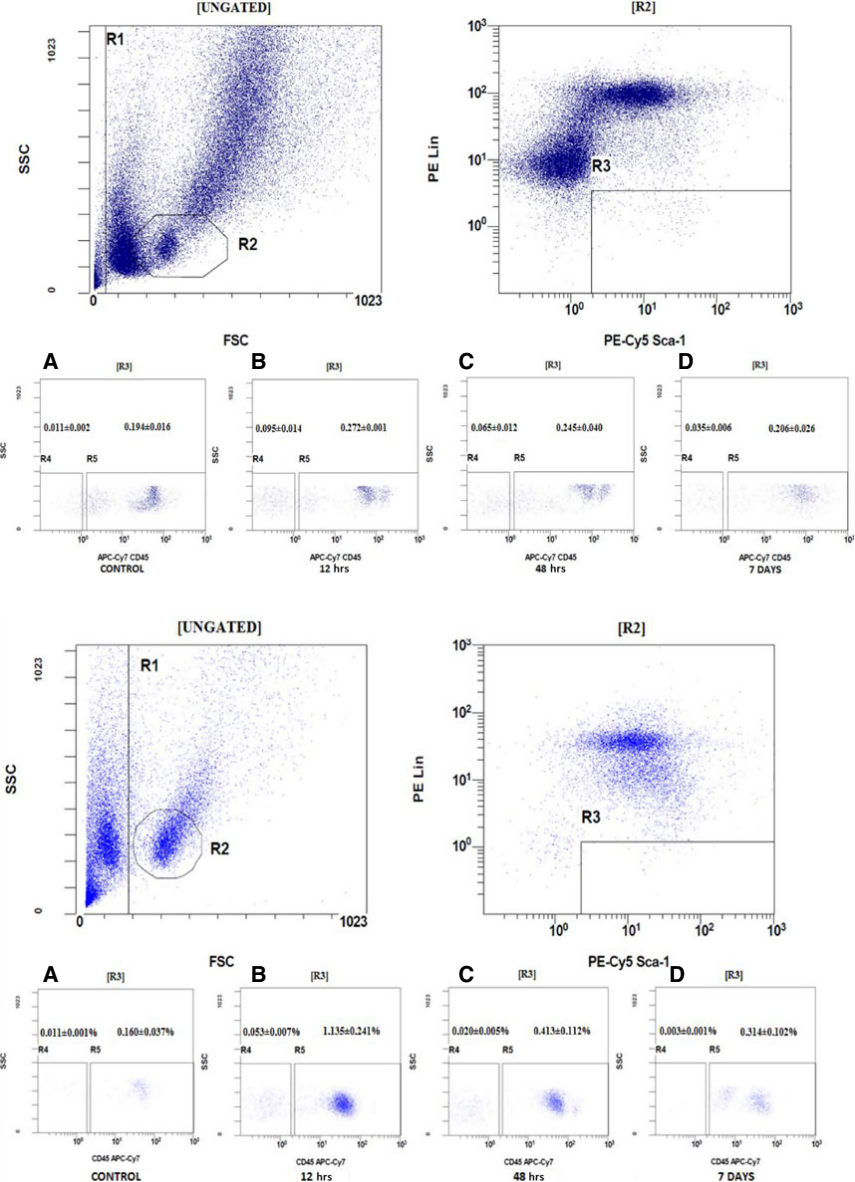
Representative example of flow cytometry analysis of the VSELs and HSCs residing in bone marrow (upper panel) and after their mobilization into peripheral blood (lower panel) in mice from group C (kainic acid 25 mg/kg bodyweight). (**A**) The number of VSELs (gate R4) *versus* HSCs (gate R5) in the control group. (**B**) The number of VSELs (gate R4) *versus* HSCs (gate R5) in subgroup II, in which the analysis was performed 12 hrs after injection of kainic acid. (**C**) The number of VSELs (gate R4) *versus* HSCs (gate R5) in subgroup IV, in which mice were analysed 48 hrs after injection of kainic acid. (**D**) The number of VSELs (gate R4) *versus* HSCs (gate R5) in subgroup V, in which the analysis was performed 7 days after injection of kainic acid.

For FACS-based studies, we selected a dose of 25 mg/kg b.w. of KA, injected once, and mice were killed at 6, 12, 24, 48 and 168 hrs (7 days) after injection. Subsequently, the numbers of VSELs and HSCs were evaluated by FACS in BM (Fig. 1 upper panel) and PB (Fig. 1 lower panel). As shown in Figure 1 upper panel, the number of Sca-1^+^Lin^−^CD45^−^ VSELs in BM increased ∼9 times and ∼6 times at 12 and 48 hrs after KA injection, respectively. Moreover, 7 days after injection, the number of VSELs was still elevated ∼3 times in comparison with control saline-injected animals. In contrast, the total number of Sca-1^+^Lin^−^CD45^+^ HSCs increased by only ∼20%.

At the same time, the number of VSELs circulating in PB had increased by ∼5 times at 12 hrs after KA injection. Also, there was a significant (∼8 times) increase in the number of circulating HSCs 12 hrs after single KA injection (25 mg/kg b.w.), and this number was still elevated by ∼3 times at 48 hrs after KA injection.

**Fig. 2:**
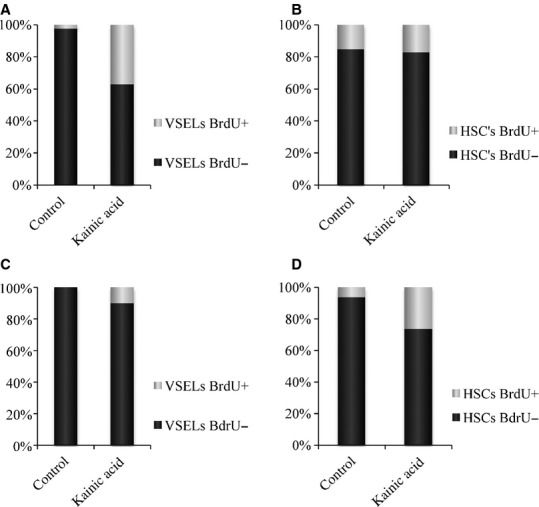
The percentage of cells that show proliferative activity and have incorporated BrdU is shown in light grey. The percentage of cells that do not proliferate (BrdU negative) is shown in dark grey. (**A**) VSELs isolated from bone marrow. (**B**) HSCs isolated from bone marrow. (**C**) VSELs isolated from peripheral blood. (**D**) HSCs isolated from peripheral blood. The data shown are combined from two independent experiments. Median values for this Figure are shown in Figure S4.

To provide further proof that quiescent VSELs enter the cell cycle in response to KA-induced brain injury, we performed BrdU labelling studies (Fig. 2). In these experiments, mice were injected five times with KA (25 mg/kg b.w.) or saline (control animals) at 3-day intervals between injections. Beginning on day 1, mice were also injected with BrdU (50 mg/kg b.w.). Figure 2A shows an increase in BrdU accumulation by BM-residing VSELs from ∼2 ± 1% to 37 ± 2%. At the same time, the number of BrdU-accumulating HSCs did not change compared with control and remained at the ∼17 ± 2% level (Fig. 2B). The elevated number of BM VSELs in the cell cycle remained detectable for a few days and returned to control values (∼2%) 1 week after KA administration (data not shown). Figure S3 shows a representative example of BrdU staining of VSELs and HSCs in BM. In parallel, we also observed an increase in the number of BrdU-accumulating VSELs circulating in PB (from 0% to ∼10 ± 2%; Fig. 2C) and an increase in the number of BrdU HSCs circulating in PB (from 5 ± 1% to ∼30 ± 3%; Fig. 2D). Figure S4 present median values which were calculated for both BM and PB analysed cells.

These experiments demonstrated for the first time that VSELs are not only mobilized into PB in response to brain injury but also that they can enter the cell cycle, proliferate and expand in BM tissue.

### Molecular analysis of BM and PB VSELs supports their BM expansion and release of neural tissue pre-committed VSELs into PB

In parallel experiments, we employed RQ-PCR analysis to evaluate the level of expression of pluripotent stem cell (PSC) genes (Oct-4, Nanog, Sox2 and Rex1) as well as changes in the expression level of genes involved in neural specification of stem cells (nestin, β3-tubulin, GFAP, Olig1 and Olig2).

**Fig. 3:**
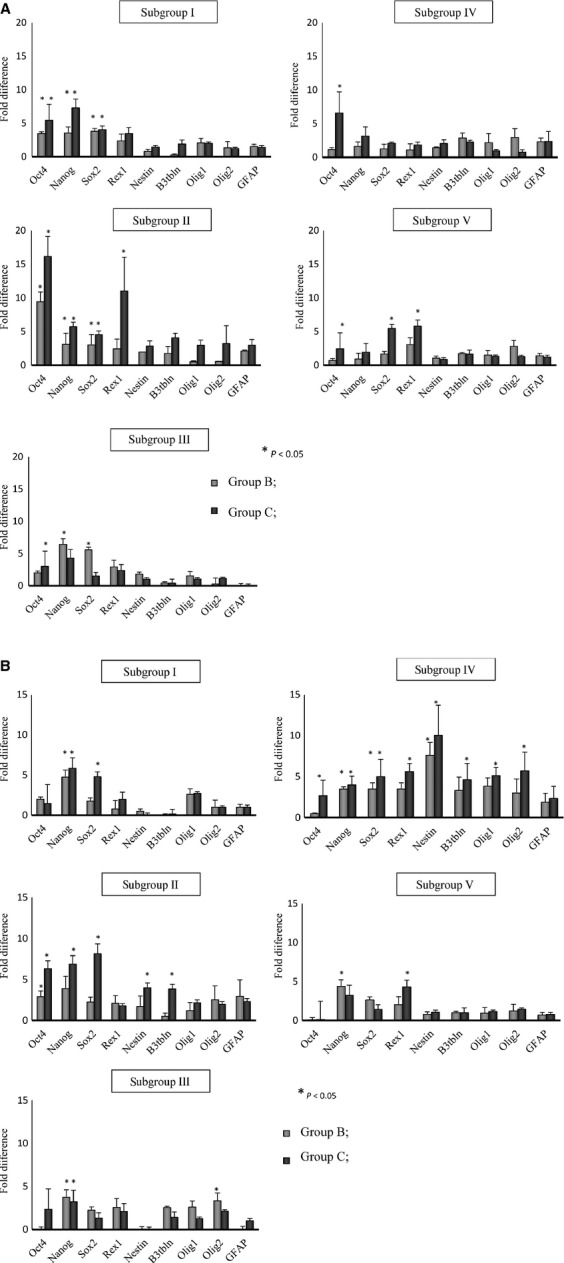
RQ-PCR analysis of the expression of selected genes in cells isolated from bone marrow (**A**) and peripheral blood (**B**) from mice in groups B and C. The cells were isolated from subgroup I at 6 hrs, subgroup II at 12 hrs, subgroup III at 24 hrs, subgroup IV at 48 hrs and subgroup V at 7 days after injection of kainic acid. Group B, animals that received kainic acid at a dose of 15 mg/kg b.w, and Group C, at 25 mg/kg b.w. Fold changes in gene expression are shown relative to expression in the control group, which was assumed to be 1.0. Combined data from three independent experiments are shown. *P < 0.05. Data from experiments with lowest dose of kainic acid (8.5 mg/kg/b.w.) are shown in Figure S5A and B.

We observed that expression of genes that regulate pluripotency increased in BM after injection of KA at doses of 15 (group B) and 25 mg/kg b.w. (group C; Fig. 3A), and Oct-4 reached its highest level 12 hrs after injection of KA. A similar pattern of gene expression was observed in PB MNC (Fig. 3B), where the expression of pluripotency genes was at its highest level 12 hrs after KA administration. However, in contrast to BM, we observed an increase in the level of genes related to neural differentiation, which were up-regulated to their highest levels in PB MNC 48 hrs after KA injection. The results obtained for the group A treated with the lowest dose of KA are shown in Figure S5A for BM and Figure S5B for PB.

These changes in gene expression support a BM expansion of VSELs that precedes subsequent mobilization into PB of cells already pre-committed into early neural progenitors.

**Fig. 4:**
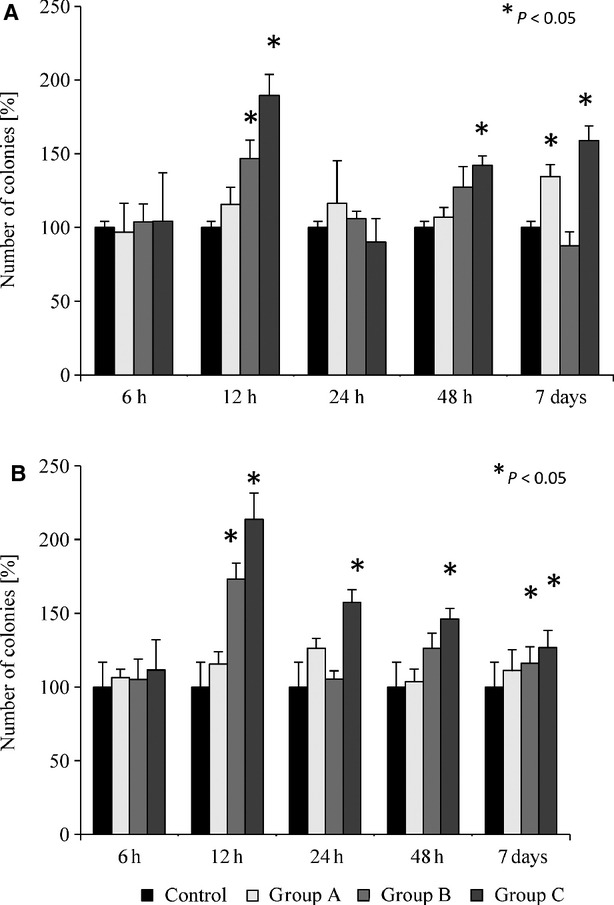
Clonogenicity of murine CFU-GM derived from the bone marrow (**A**) and peripheral blood (**B**) of animals treated with various doses of kainic acid. Group A animals received 8.5 mg/kg kainic acid; group B animals received 15 mg/kg bw kainic acid; group C animals received 25 mg/kg bw kainic acid. Combined data from three independent experiments are shown. *P < 0.05.

As shown in Figure 4, we also observed a slight parallel increase in the number of clonogenic CFU-GM progenitors, particularly 12 hrs after KA administration, both in BM (Fig. 4A) and PB (Fig. 4B). We also evaluated the number of clonogenic BFU-E in BM and PB of mice treated with KA and observed an increase in the number of BFU-E progenitors in PB 12 hrs after KA administration; however, at the same time-point their number in BM remained unchanged (data not shown).

### Immunohistochemical and molecular analysis of VSELs isolated by fluorescence- activated cell sorting from BM and PB

**Fig. 5:**
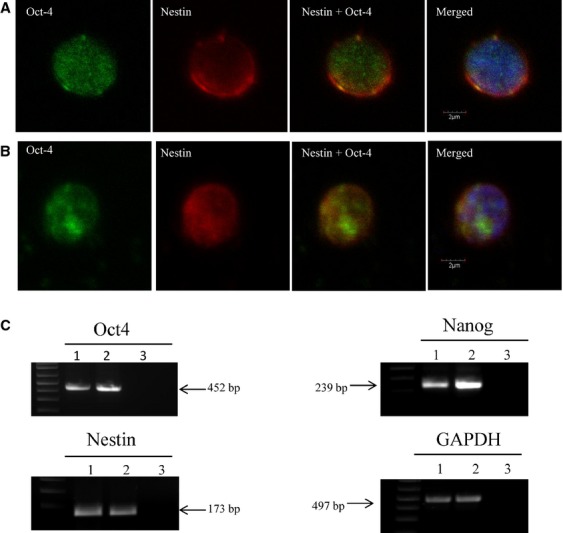
(**A** and **B**) Representative images of immunohistochemical analysis of VSELs isolated by fluorescence-activated cell sorting from bone marrow and peripheral blood after KA administration (25 mg/kg b.w). (**A**) BM-derived Sca1^+^/Lin^−^/CD45^−^ cells express Oct-4 and nestin. (**B**) PB-derived Sca1^+^/Lin^−^/CD45^−^ cells express Oct-4 and nestin. All images were taken under a Plan Apo 60XA/1.40 oil objective (Nikon). Staining was performed on cells isolated from three independent sorts with similar results. (**C**) RT-PCR analysis of the expression of selected pluripotency genes: Oct4, Nanog, and the neural marker nestin in cells sorted from bone marrow of control and kainic acid-treated mice (25 mg/kg b.w). Lane 1, control mouse; Lane 2, kainic acid-treated mouse; Lane 3, H_2_O negative control. GAPDH expression served as loading control. A representative analysis is shown.

To better characterize VSELs residing in BM and circulating in PB of mice exposed to KA (25 mg/kg b.w.), small Sca-1^+^Lin^−^CD45^−^ cells were sorted from BM and PB after long-term exposure to KA (the highest dose repeated five times). As shown in Figure 5A and B, we were able to demonstrate the presence of Oct-4 and nestin at the protein level in BM- and PB-derived VSELs. Expression of Oct-4 and nestin was subsequently confirmed in BM-derived VSELs by conventional RT-PCR (Fig. 5C).

## Discussion

The salient observation of this report is that a quiescent population of BM-residing VSELs responds by expansion to exposure to the neurotoxin KA. In many previous models of tissue/organ injuries, such as heart infarct, stroke, skin burns, sodium iodide-induced retinal damage or Crohn's disease, we and others have demonstrated that VSELs can be mobilized in stress situations from BM into PB, assuming that BM is the primary source of these cells [11,13,24–26]. In this report, we provide evidence that a quiescent population of BM-residing VSELs indeed proliferates and expands in BM in response to neurotoxic damage, and at the same time, undergoes specification into the neural lineage, as revealed by RQ-PCR analysis of neural gene expression and immunohistochemical staining. This process seems to precede subsequent egress/mobilization of these cells from BM into PB.

The field of regenerative medicine is searching for stem cells that could be employed to regenerate damaged brain [27–32]. Data from mouse and rat have shown that brain damage induced by stroke and hypoxia leads to increased proliferation of neural precursors that are located in the SVZ of the lateral ventricles and olfactory bulb as well as in the SGZ of the DG in the hippocampus [4–6,33]. These anatomical areas contain ‘neurovascular niches’ composed of neuroblasts, astrocytes and neural stem cell/neuronal precursor cells that reside in proximity to a rich microvascular network [34]. However, it is obvious at this point that brain cannot be considered as a source of autologous neural stem cells for therapeutic purposes.

However, mice with cyclin D2 deficiency have a severely reduced number of stem cells in all these classical stem cell locations yet possess normally developed brains, which suggests the involvement in neural tissue homoeostasis of stem cells that reside in other locations [8,9,35,36]. One possibility is that these cells can translocate to the brain *via* PB from other organs or tissues, most likely from the BM. While direct evidence is still missing on whether stem cells for brain tissue can be derived from a population of BM-derived primitive stem cells circulating in blood (*i.e*., VSELs), a detectable level of donor-derived chimerism in brain tissue has been observed in patients after haematopoietic transplantation, which supports this tempting possibility [37]. To explain these data, transplant-derived neural cells identified in these patients could be descendants of primitive pluripotent/multipotent stem cells (*e.g*., VSELs) that were infused into the patients along with the haematopoietic graft.

In our experiments, KA-induced brain damage leads to an increase in the number of circulating VSELs and HSCs in PB. This observation is in agreement with several models of tissue/organ injuries and stress, including stroke, where it has been demonstrated that there is an increase in the number of VSELs, HSCs, endothelial progenitors and MSCs circulating in PB [12,13,38]. Nevertheless, an important question still remains: are these cells merely a sign of tissue damage or are they being mobilized to contribute to the regeneration process? The fact that in our study developmentally early cells circulating in PB express several neural markers (*e.g*., nestin, β3-tubulin, GFAP, Olig1 and Olig2) demonstrates their commitment into the neural lineage. We envision that cells circulating in PB could be potentially involved in repairing minor tissue damage; however, these cells are obviously ineffective in repairing more extensive necrotic areas [39]. Their ineffectiveness could result from the fact that massive tissue damage induces a highly proteolytic environment (as the result of attracting granulocytes, which secrete proteolytic enzymes) that disrupts chemotaxis and homing of circulating stem cells to the damaged organ. A more robust contribution of circulating stem cells in brain regeneration is also probably hampered by the presence of the blood–brain barrier [40].

We also have to consider that stem cells circulating in PB are also a known source of several growth factors, cytokines and bioactive lipids as well as extracellular vesicles that may indirectly protect damaged tissues from undergoing apoptosis and promoting vascularization [41]. Such effects have been very well demonstrated for HSCs and MSCs; however, further studies are needed to see which potential paracrine effects are released from VSELs circulating in PB.

Very small embryonic-like stem cells express several PSC markers (*i.e*., Oct-4, Nanog and SSEA), and true expression of the pluripotency transcription factor Oct-4 in these cells was confirmed by demonstrating the unmethylated state of the Oct-4 promoter and its epigenetic histone code, which is characteristic of transcriptionally active DNA [17,18]. Furthermore, as recently reported, most of the homoeodomain-containing developmental transcription factors in VSELs are repressed by specific epigenetic marks, called bivalent domains, which represent a state of the DNA structure characteristic of PSCs, where transcriptionally antagonistic histone codes physically co-exist within the same promoter [42].

Murine as well as human Oct-4^+^ VSELs do not easily proliferate spontaneously *in vitro* if cultured alone, and the quiescence of these cells is regulated by genomic imprinting through DNA methylation, which is an epigenetic program that ensures the parent-specific mono-allelic transcription of specific developmentally important genes [17,18]. This, however, does not preclude the possibility that they may become activated in situations of hypoxic stress in damaged tissues, and we are currently exploring this possibility.

In this report, we show for the first time that despite epigenetic changes that keep these cells under control and prevent their proliferation, VSELs may expand in BM in response to organ damage, as seen in a model of KA-toxic brain injury. This supports their potential role in tissue/organ regeneration; however, more direct evidence is needed. Based on this possibility, we propose that VSELs freshly isolated from BM, PB or UCB and pre-committed to the neurological lineage in *ex vivo* cultures could be a potential source of cells for regeneration of the CNS. Ongoing studies in our laboratories will address this attractive possibility to determine whether these cells could be efficiently employed in the clinic or whether they are merely developmental remnants found in the BM that cannot be harnessed effectively for regeneration.

In the light of the most recent work from Dr. Vacanti's group, it is also possible that VSELs are generated in BM tissue in response to stress related to the toxic effects of KA [43]. The coming years will bring definitive answers to these questions.

## Supporting information

**Figure S1** Schematic overview of experiments.

**Figure S2** A representative example of the analysis of morphological changes in the hippocampus by haematoxylin/eosin staining.

**Figure S3** A representative example of flow cytometry analysis of BrdU accumulation in VSELs and HSCs in the bone marrow of mice exposed to KA and in control animals.

**Figure S4** Upper panel.

**Figure S5** RQ-PCR analysis of the expression of selected genes in cells isolated from bone marrow (A) and peripheral blood (B) from mice in groups A and control group.
