# Supplementary figures and images for: Evidence that the population of quiescent bone marrow-residing very small embryonic/epiblast-like stem cells (VSELs) expands in response to neurotoxic treatment
Source: J Cell Mol Med. 2014 Jun 4;18(9):1797–806. doi: 10.1111/jcmm.12315 (PMC4162847; doi:10.1111/jcmm.12315)

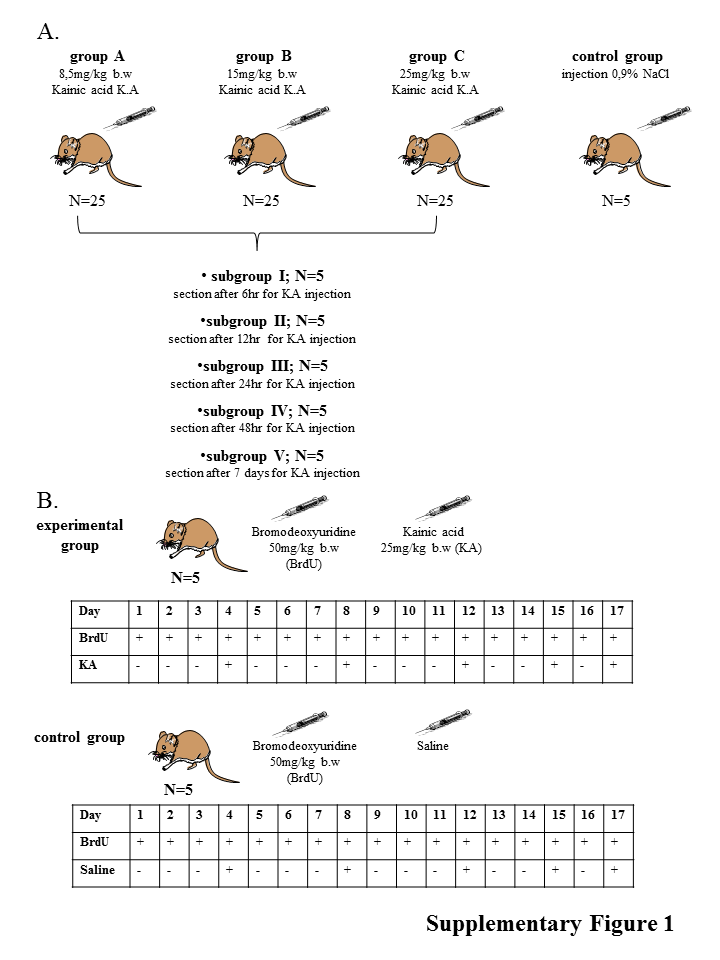

Supplement: Supplementary file 1 — Figure S1 Schematic overview of experiments. [file jcmm0018-1797-SD1.tif]

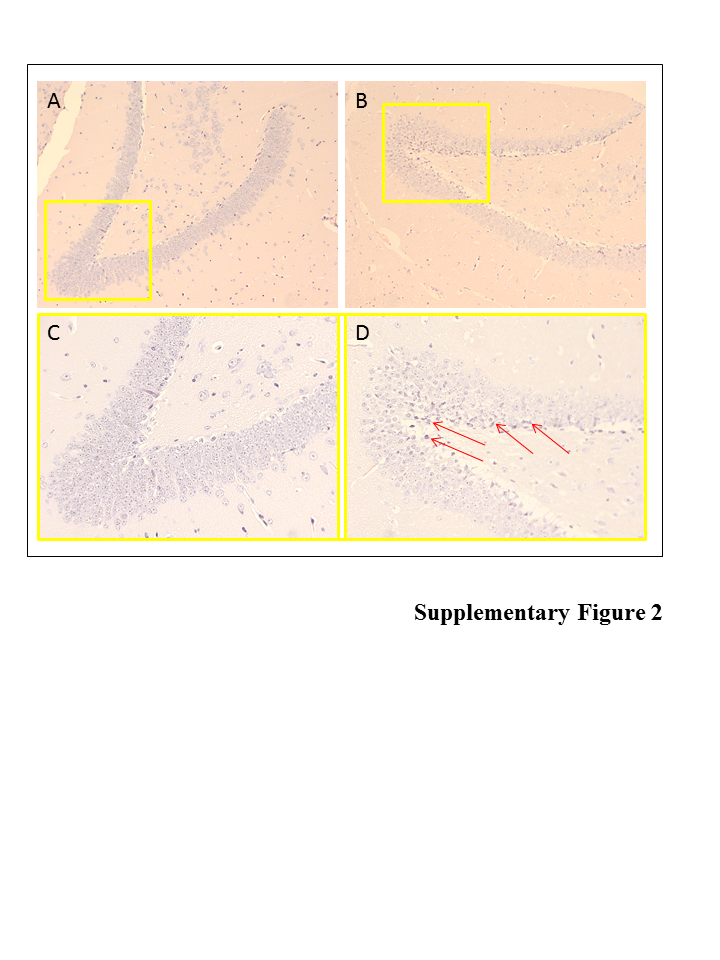

Supplement: Supplementary file 2 — Figure S2 A representative example of the analysis of morphological changes in the hippocampus by haematoxylin/eosin staining. [file jcmm0018-1797-SD2.tif]

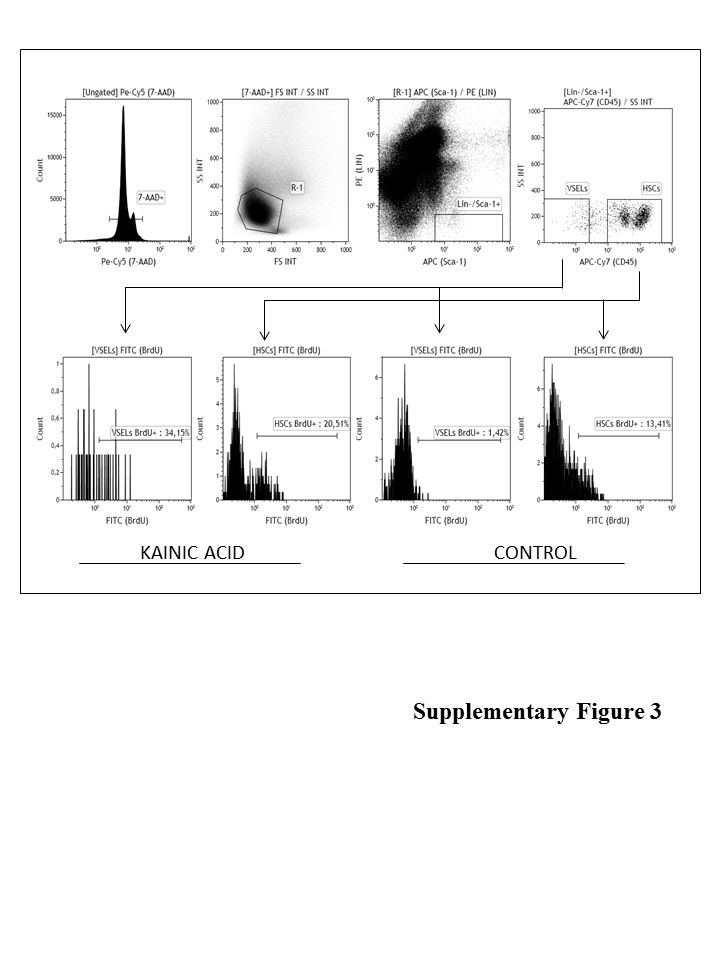

Supplement: Supplementary file 3 — Figure S3 A representative example of flow cytometry analysis of BrdU accumulation in VSELs and HSCs in the bone marrow of mice exposed to KA and in control animals. [file jcmm0018-1797-SD3.tif]

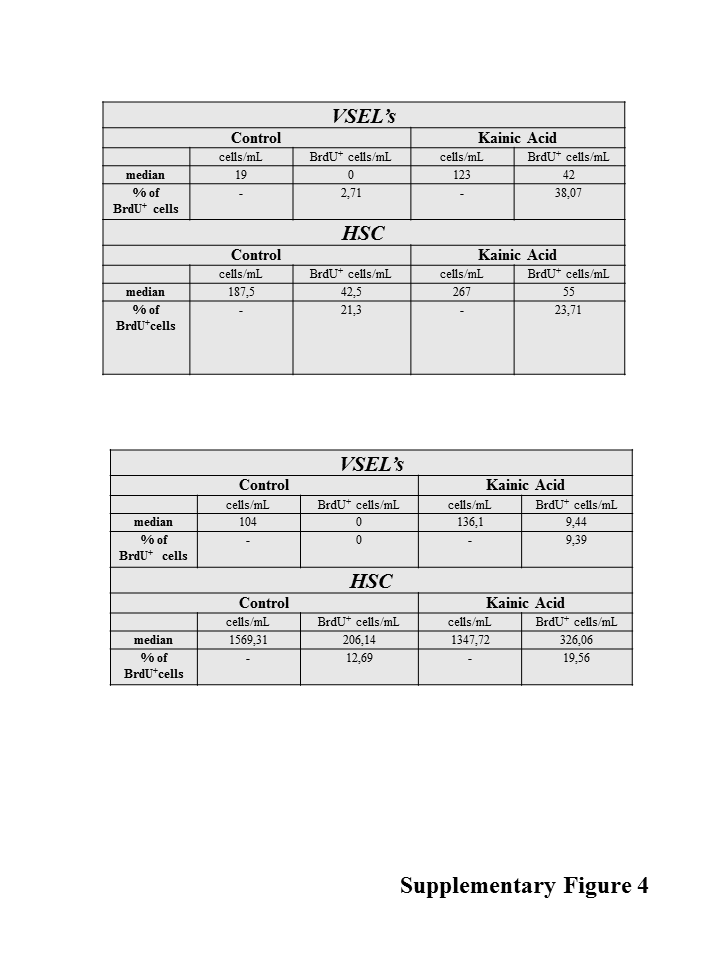

Supplement: Supplementary file 4 — Figure S4 Upper panel. [file jcmm0018-1797-SD4.tif]

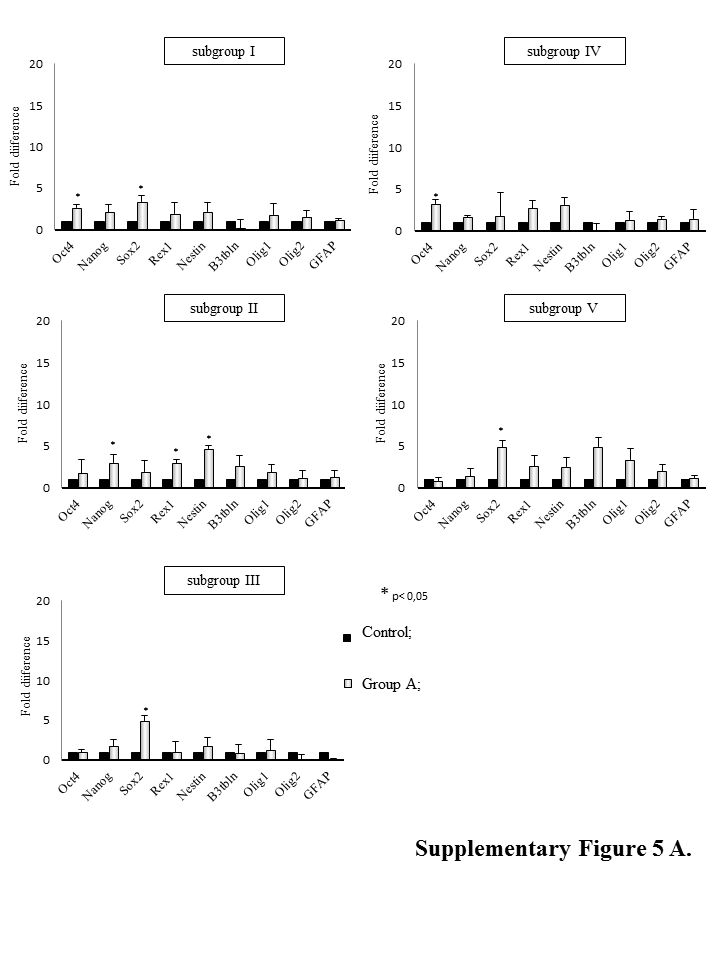

Supplement: Supplementary file 5 — Figure S5 RQ-PCR analysis of the expression of selected genes in cells isolated from bone marrow (A) and peripheral blood (B) from mice in groups A and control group. [file jcmm0018-1797-SD5.tif]

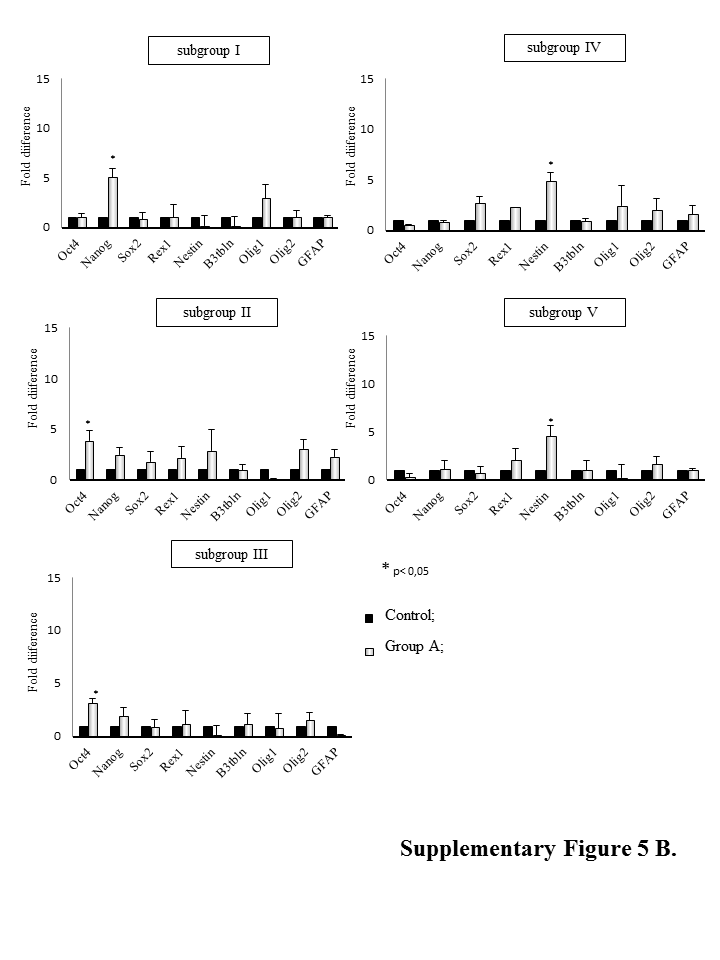

Supplement: Supplementary file 6 — Figure S5 RQ-PCR analysis of the expression of selected genes in cells isolated from bone marrow (A) and peripheral blood (B) from mice in groups A and control group. [file jcmm0018-1797-SD6.tif]
